# Poorer Prognosis of Idiopathic Pleuroparenchymal Fibroelastosis Compared with Idiopathic Pulmonary Fibrosis in Advanced Stage

**DOI:** 10.1155/2018/6043053

**Published:** 2018-08-13

**Authors:** Makoto Shioya, Mitsuo Otsuka, Gen Yamada, Yasuaki Umeda, Kimiyuki Ikeda, Hirotaka Nishikiori, Koji Kuronuma, Hirofumi Chiba, Hiroki Takahashi

**Affiliations:** Department of Respiratory Medicine and Allergology, Sapporo Medical University School of Medicine, South-1, West-16, Sapporo 060-8543, Japan

## Abstract

**Objective:**

Idiopathic pleuroparenchymal fibroelastosis (IPPFE) is a rare disease characterized by predominant upper lobe pulmonary fibrosis of unknown etiology. However, the prognosis of IPPFE has not been discussed. We investigated the clinical characteristics and prognostic factors of IPPFE and idiopathic pulmonary fibrosis (IPF).

**Methods:**

We performed a retrospective cohort study on 375 consecutive idiopathic interstitial pneumonia patients between April 2004 and December 2014. Among them, we diagnosed IPPFE and IPF patients using high-resolution computed tomography radiological criteria.

**Results:**

Twenty-nine IPPFE patients (9 males, 20 females) and 67 IPF patients (54 males, 13 females) were enrolled. IPPFE patients were significantly more likely to be females and nonsmokers and had lower body mass index, lower values of predicted percentage of forced vital capacity (%FVC), and a higher residual volume-to-total lung capacity ratio than IPF patients. Survival analysis revealed that they had significantly poorer prognosis than IPF patients in GAP (gender, age, and physiology) stages II + III. %FVC and GAP index independently predict mortality in patients with IPPFE.

**Conclusions:**

Patients with IPPFE showed poorer prognosis in the advanced stage than patients with IPF. %FVC and GAP index are independent predictors of survival in patients with IPPFE.

## 1. Introduction

Pleuroparenchymal fibroelastosis (PPFE) was initially described as upper lobe pulmonary fibrosis of unknown etiology by Amitani et al. [1]. In 2013, idiopathic PPFE (IPPFE) was newly listed as a rare idiopathic interstitial pneumonia (IIP) in the IIP classification [2]. The clinical characteristics of IPPFE show a history of recurrent pneumothorax, recurrent infection, and weight loss [3]. High-resolution computed tomography (HRCT) of IPPFE patients shows upper lobe involvement with dense subpleural consolidation, architectural distortion, and upper lobe volume loss [4]. However, the prognosis of IPPFE remains unclear. Oda et al. [5] demonstrated that usual interstitial pneumonia (UIP) with IPPFE is clinically different from UIP/idiopathic pulmonary fibrosis (IPF) and that the former tended to have a poor prognosis. Watanabe et al. [6] reported that IPPFE patients show a rapid decrease in forced vital capacity (FVC). From these reports, we speculated that IPPFE patients have a poorer prognosis than IPF patients. IPPFE is generally diagnosed via histological examination of the lung tissue. However, surgical lung biopsy is a high-risk and invasive diagnostic procedure for IIPs that can cause severe complications. Because HRCT findings of IPPFE are distinctive from those of the other IIPs [7–9], we consider HRCT to be sufficient to diagnose IPPFE.

Here, we identified patients with IPPFE using HRCT radiological criteria and compared the clinical profiles, blood gas analysis, pulmonary function tests, and prognosis between IPPFE and IPF. We elucidated that the clinical features of IPPFE were different from those of IPF, and IPPFE showed a poorer prognosis compared with IPF in advanced stages.

## 2. Patients and Methods

### 2.1. Study Design

We performed a retrospective cohort study of 375 consecutive IIPs patients at Sapporo Medical University Hospital between April 2004 and December 2014. This study was approved by the Institutional Review Board of Sapporo Medical University Hospital (#282–1052, approved on October 17, 2015), and the need for informed consent from the patients was waived because of the retrospective nature of the study. Of the 375 subjects, IPPFE and IPF patients were selected and compared with regard to their clinical parameters and survival. By reviewing their radiological findings, laboratory data, clinical symptoms, occupational history, living environment, and contact history to bird and other potential antigen, we excluded patients with cardiovascular, infectious, neoplastic, or allergic diseases; those presenting with a lung disease due to exposure to occupational dust, such as asbestosis; and those presenting with tuberculosis, sarcoidosis, hypersensitivity pneumonia, collagen vascular diseases, or a history of chest operations.

### 2.2. IPPFE and IPF Diagnoses

Patients with pathologically diagnosed IPPFE or radiologically diagnosed IPPFE were selected and enrolled into this study. IPPFE patients were diagnosed by four expert pulmonologists, without the knowledge of the clinical information of the patients, according to the HRCT radiological criteria for IPPFE diagnosis [10] as follows: definite IPPFE, pleural thickening with associated subpleural fibrosis is upper lobe predominant with less marked or no involvement of the lower lobes; consistent with IPPFE, pleural thickening with associated subpleural fibrosis is upper lobe dominant but the distribution of these changes is not upper lobe dominant or features of coexisting upper lobe pleural thickening are present elsewhere but the distribution of all HRCT findings is evidently upper lobe dominant; inconsistent with IPPFE, lacking the requisite features described earlier. If there are HRCT findings in the middle or lower lobe, these findings are relatively limited compared to the PPFE lesion in the upper lobe and discontinuous from the PPFE lesion. HRCT findings were reviewed two or three times throughout the clinical course. Patients were only included when they were considered definite or consistent with IPPFE. In addition, the histological criteria for PPFE [11] were applied to the cases where the patients underwent surgical biopsy.

In addition, IPF patients were diagnosed according to the 2011 American Thoracic Society (ATS)/European Respiratory Society (ERS)/Japan Respiratory Society (JRS)/Latin American Thoracic Association (ALAT) IPF statement [12].

### 2.3. Clinical and Radiological Review

All subjects were reviewed in terms of their clinical information, radiological data, pulmonary function test (PFT) results, and laboratory data from the medical records on the date of the first visit to our hospital. We evaluated HRCT findings and the ratio of the anteroposterior diameter of the thorax (APDT) to the transthoracic diameter of the thorax (TDT) on the HRCT using published criteria [12–14]. In the PFTs, we examined the annual changes in the parameters, including FVC, total lung capacity (TLC), residual volume (RV), and diffusing capacity of the lung for carbon monoxide (DLco).

Survival analysis was performed from the date of the first visit to our hospital. The overall survival of the IPPFE and IPF groups was compared using the GAP (gender, age, and physiology) index and staging system [15].

### 2.4. Statistical Analysis

All data were expressed as the mean ± standard deviation (SD) or 95% confidence interval. Differences between the two groups were assessed using the Mann–Whitney *U* test. A chi-square test or Fisher's exact test was used to compare categorical data. Survival analysis was performed using the Kaplan–Meier method, and the log rank test and the generalized Wilcoxon test were used to compare the survival curves. The univariate Cox proportional hazard model was used to examine the association of the selected variables with survival. Variable selections in multivariate analysis were performed with respect to age, and variables with a *p* value less than 0.05 were analyzed by the univariate analysis. Selected variables were age, sex, clubbed finger, history of pneumothorax, GAP index, predicted percentage of forced vital capacity (%FVC), predicted percentage of diffusion capacity (%DLco), alveolar-arterial oxygen difference (A-aDO_2_), and Krebs von den Lungen (KL-6). All tests were performed at a significance level of *p* < 0.05, and statistical analyses were performed using IBM SPSS Statistics (version 22; IBM Corp., NY, USA).

## 3. Results

### 3.1. IPPFE and IPF Patients

Among the 375 patients with IIPs, we identified 29 IPPFE patients who met the radiological criteria for IPPFE. Of these, radiological findings of 2 patients indicated definite IPPFE and those of 27 patients were consistent with IPPFE. Three patients underwent surgical lung biopsy and fulfilled the histological criteria for PPFE. In contrast, we identified 67 IPF patients who met the ATS/ERS/JRS/ALAT criteria [12]; of these, eight patients underwent surgical lung biopsy and fulfilled the histological criteria for IPF.

### 3.2. Demographic Features

The baseline features are summarized in Table 1. In the IPPFE group, 9 were male and 20 were female (mean age ± SD: 69 ± 7.3 years old). Patients with IPPFE were significantly more likely to be female and nonsmokers, have smoked for fewer pack-years, had a lower body mass index, and had a lower APDT-to-TDT ratio than IPF patients. Three patients in the IPPFE group had a history of pneumothorax. The symptoms in the IPPFE group were cough (*n*=13; 45%), dyspnea on exertion (*n*=7; 24%), clubbed finger (*n*=6; 21%), and fine crackles (*n*=25; 86%) at the first visit, as well as in the IPF group. A higher percentage of the IPPFE group showed presenting symptom (72% versus 48%) and a lower percentage of mass screening (28% versus 52%) for the reasons of the first medical examination compared with the IPF group (*p*=0.03).

### 3.3. PFT, BGA, Serum Biomarkers, and HRCT

The IPPFE group showed significantly lower values of the predicted percentage of FVC (%FVC) and higher values of forced expiratory volume percent in one second (FEV_1_/FVC), RV-to-TLC ratio (RV/TLC), and PaCO_2_ than the IPF group (Table 2). The IPPFE group had significantly lower values of surfactant protein (SP)-A and Krebs von den Lungen (KL)-6, as well as a lower positive rate of serum KL-6 than the IPF group. Two patients (anti-CCP antibody and anti-dsDNA antibody) in the IPPFE group and four patients (anti-SS-A antibody, anti-Scl-70 antibody, and anti-CCP antibody) in the IPF group showed positivity of specific autoantibodies.

The HRCT findings of IPPFE patients are summarized in Table 3. Totally, all 29 patients with IPPFE demonstrated marked subpleural consolidation in the bilateral upper lobes. Nine patients (31%) had accompanying honeycombing and 27 (93%) had traction bronchiectasis in the upper lobes. Moreover, 10 patients (34%) demonstrated a definite UIP pattern, seven (24%) a possible UIP pattern, and 10 (35%) a nonspecific interstitial pneumonia (NSIP) pattern in the middle or lower lobes.

### 3.4. Outcome, Events, and Survival

Eleven patients (38%) in the IPPFE group and 29 (43%) in the IPF group died during the observation period. In the IPPFE group, four patients died of chronic respiratory failure, four of acute exacerbation, and three of other causes, namely, pneumonia, pulmonary embolism, and suicide (Table 4). Nineteen patients in the IPPFE group and four in the IPF group had pneumothorax or pneumomediastinum. Eight (73%) of IPPFE patients who died had a history of pneumothorax or pneumomediastinum. Moreover, we had one patient with prolonged pneumothorax after surgical lung biopsy and one patient with prolonged pneumothorax leading to acute exacerbation. Two of the nine IPPFE patients treated with oral corticosteroids showed improvements on HRCT. Those who showed restrictive ventilatory defects did not show improvements in the PFT results. One of the 10 IPPFE patients treated with pirfenidone maintained their %FVC values for over a year whereas the others did not during the observation period.

Seventeen patients in the IPPFE group and 57 patients in the IPF group underwent PFT twice a year (mean interval, 1.03 ± 0.22 years; Table 5). The IPPFE group showed significantly lower values of annual changes in FVC and TLC than the IPF group.

**Figure 1:**
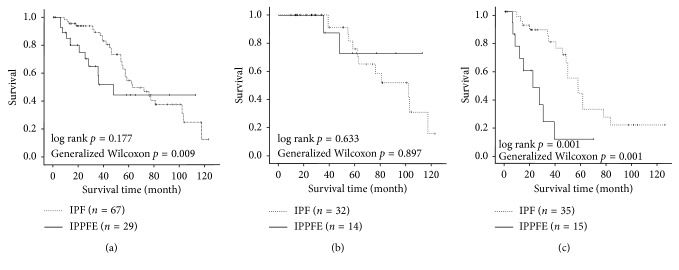
Kaplan–Meier survival curves. (a) Overall, the IPPFE group had significantly worse survival than the IPF group (log rank *p*=0.177, generalized Wilcoxon *p*=0.009). (b) In the GAP stage I, there was no significant difference between the IPPFE and IPF groups in terms of survival. (c) In the GAP stages II + III, the IPPFE group had significantly worse survival than the IPF group (log rank *p*=0.001, the generalized Wilcoxon *p*=0.001).

The mean lengths of observation period in the IPPFE and IPF groups were 64 ± 10 and 74 ± 5.3 months, respectively. The survival analysis showed that the IPPFE group had significantly worse survival than the IPF group (log rank *p*=0.177, the generalized Wilcoxon *p*=0.009; Figure 1(a)). Thereafter, based on the GAP index and staging system, IPPFE and IPF patients were classified into two stages: GAP stage I and GAP stages II + III. In the GAP stage I, there was no significant difference in survival between the IPPFE and IPF groups (Figure 1(b)). In contrast, the IPPFE group showed significantly worse survival than the IPF group in the GAP stages II + III (log rank *p*=0.001, the generalized Wilcoxon *p*=0.001; Figure 1(c)).

### 3.5. Evaluation of the Prognostic Factors in IPPFE Patients

The univariate Cox proportional hazard model demonstrated that the following variables had statistically significant effects on survival: female, clubbed finger, pack-years of smoking, history of pneumothorax, GAP index, GAP stage, %FVC, predicted percentage of TLC (%TLC), %DLco, predicted percentage of diffusing capacity divided by the alveolar volume (%DLco/VA), A-aDO_2_, KL-6, ΔFVC, and ΔTLC (Table 6). In addition, the frequency of lower lobe involvement in IPPFE patients showed no significant effect on survival (Table 6).

The multivariate Cox proportional hazard model demonstrated that GAP index (HR: 2.510, *p*=0.010) and %FVC (HR: 0.903, *p*=0.017) independently predict mortality in patients with IPPFE.

## 4. Discussion

The prognosis of IPPFE has not been fully investigated. In the present study, we elucidated the differences between IPPFE and IPF with respect to the clinical characteristics, laboratory results, annual changes of pulmonary functions, and survival. The prognosis of patients with IPPFE was significantly poorer than that of patients with IPF, and %FVC and GAP index were significantly associated with poorer prognosis of IPPFE. As per our knowledge, there was only one prognostic article, which stated that the survival time of patients with PPFE with UIP pattern tended to be shorter than that of patients with IPF/UIP [5]. In our study, there were differences in PFT at the time of diagnosis, with significantly lower %FVC and higher RV/TLC in the IPPFE group than in the IPF group; therefore, we compared the prognosis between the two groups using the GAP index and staging system. The GAP index and staging system is a validated risk prediction model for mortality among patients with IPF [15]. We demonstrated that IPPFE patients had significantly poorer prognosis than all IPF and those at the GAP stage II + III. These results may indicate that IPPFE is more deteriorative than IPF in the advanced stage. Furthermore, GAP index was a significant prognostic factor for patients with IPPFE. These results suggest that GAP index may also predict mortality in patients with IPPFE.

Approximately 60% of patients with IPPFE with a coexisting UIP pattern in the lower lobes were observed in the present study. Despite IPPFE involving UIP lesions in the lower lobe, the clinical features and prognosis of IPPFE and IPF were completely different. Three previous studies reported rates of the presence of a coexisting UIP pattern to be 54% [7], 43% [10], and 75% [16]. Thus, a coexisting UIP pattern in the lower lobes may be characteristic in IPPFE patients. In contrast, the cause of death in IPPFE patients included acute exacerbations, which are also found in IPF. As IPPFE progresses, not only chronic respiratory failure but also acute exacerbations may be involved [17]. We speculate that UIP lesions in the lower lobes are possibly the origin of these acute exacerbations.

Although we diagnosed IPPFE using the radiological criteria, the clinical features of patients with IPPFE diagnosed using radiological criteria were thought to be consistent with the characteristics of pathologically diagnosed patients with PPFE previously reported [6, 11, 16–19]. IPPFE diagnosis is generally made via histological examination of the lung tissue. However, surgical lung biopsy is a high-risk and invasive diagnostic procedure for IIPs that can cause severe complications. Postoperative pneumothorax is a frequent complication in the surgical lung biopsy of patients with PPFE [10]. Most patients with IPPFE in this study were unable to undergo surgical lung biopsy because of the advanced stage during diagnosis. Camus et al. [7] proposed that surgical lung biopsy is unnecessary for cases that clinically and radiologically meet IPPFE characteristics. Because the radiological characteristics of PPFE are very distinctive compared with those of other ILDs, the possibility of radiological diagnosis using HRCT has been proposed [7–9]. We think that the radiological criteria reported by Reddy et al. [10] are appropriate for the clinical diagnosis of IPPFE.

Eighteen of the 29 (62.1%) patients were treated with corticosteroids or pirfenidone after diagnosis. Corticosteroids were often used to treat patients in this study with acute or subacute progressive diseases. These patients showed improvements on HRCT but not in the PFT results. Only one of the nine (11%) patients treated with pirfenidone was able to maintain their %FVC for over a year. These treatments were not previously considered as effective for IPPFE patients who reportedly have similar characteristics [17]. However, pirfenidone was effective for treating IPPFE combined with UIP in the lower lobes [20]. Pirfenidone may be an effective drug for IPPFE patients with a UIP lesion because it reduces the progression of fibrotic changes. Further research is warranted to examine the efficacy of pirfenidone in IPPFE patients.

Here, patients with IPPFE showed significantly lower serum SP-A and KL-6 levels than patients with IPF. According to two previous reports on the same nine IPPFE cases [18, 21], the serum SP-D level was higher than the normal value in all cases; conversely, the KL-6 level increased in only three cases. It is still unclear why the serum SP-D level is highly elevated, whereas the KL-6 level is normal or slightly higher than the normal range in most IPPFE patients. Sato et al. [21] reported that their immunohistochemical analysis for SP-D demonstrated that the hyperplastic epithelial cells in the upper lobes were more strongly stained than those in the lower lobes, although they found that KL-6 was homogeneously stained in the lung tissues of both the upper and lower lobes. These differences may contribute to serum biomarker elevations in IPPFE.

Our study had several limitations. First, this was a retrospective cohort study conducted at only a single institute. Second, we did not perform pathological assessments; however, we carefully considered other etiologies of upper lobe fibrosis, such as occupational dust exposure, infection, autoimmune disease, sarcoidosis, and hypersensitivity pneumonia [3]. Camus et al. [7] proposed that the differential diagnosis can be resolved by reviewing earlier imaging; searching for extrathoracic involvement, which is typically absent in IPPFE; and performing appropriate laboratory tests for infections and connective tissue diseases. In our study, we performed these examinations and could discriminate other types of upper lobe fibrosis.

## 5. Conclusions

The clinical features of IPPFE diagnosed using HRCT were consistent with the characteristics of pathologically diagnosed patients with PPFE and differed from those of IPF. IPPFE prognosis is worse than IPF prognosis. The clinical diagnostic criteria for IPPFE still need to be established.

## Figures and Tables

**Table 1:** The baseline characteristics of IPPFE and IPF patients.

	IPPFE	IPF	*p* value
	(*n*=29)	(*n*=67)	
Sex M/F	9/20	54/13	<0.01
Age (years)	69 ± 7.3	69 ± 7.8	0.81
Smoker/nonsmoker	11/18	56/11	<0.01
Number of pack-years	10.2 ± 20.0	34.7 ± 27.0	<0.01
History of pneumothorax	3 (10%)	1 (1.4%)	0.08
Family history of IPs	3 (10%)	10 (14.9%)	0.40
BMI (kg/m^2^)	20.1 ± 3.25	24.1 ± 2.97	<0.01
Thoracic dimensions			
APDT/TDT (%)	59.9 ± 6.0	65.4 ± 5.2	<0.01
Symptoms			
Cough	13 (45%)	20 (30%)	0.16
Dyspnea on exertion	7 (24%)	13 (19%)	0.60
Clubbed finger	6 (21%)	16 (24%)	0.73
Fine crackles	25 (86%)	65 (97%)	0.07
First medical examination			
Symptomatic	21 (72%)	32 (48%)	0.03
Mass screening	8 (28%)	35 (52%)	

Data presented as the mean ± SD or numbers. IP = interstitial pneumonia; BMI = body mass index; APDT = anteroposterior diameter of the thorax; TDT = transverse diameter of the thorax.

**Table 2:** The baseline physiological characteristics and laboratory results of IPPFE and IPF patients.

	IPPFE	IPF	*p* value
	(*n*=29)	(*n*=67)	
FVC %pred (%)	71.8 ± 19.4	88.2 ± 18.5	<0.01
FEV_1_/FVC (%)	86.0 ± 13.2	82.8 ± 7.29	<0.01
TLC %pred (%)	74.3 ± 14.2	78.3 ± 16.3	0.21
RV %pred (%)	76.9 ± 18.5	68.1 ± 19.3	0.10
RV/TLC (%)	40.2 ± 9.15	29.6 ± 6.90	<0.01
DLco %pred (%)	52.4 ± 12.9 (*n*=24)	51.7 ± 14.8 (*n*=61)	0.55
SpO_2_ (%)	96.6 ± 1.50	96.3 ± 1.58	0.42
A-aDO_2_ (Torr)	12.2 ± 11.1 (*n*=27)	13.0 ± 7.45 (*n*=62)	0.57
PaCO_2_ (Torr)	43.4 ± 3.57 (*n*=27)	40.8 ± 3.40 (*n*=62)	<0.01
ANA (<320/≥320)	24/5	61/6	0.30
SP-A (ng/ml)	61.2 ± 22.3	84.2 ± 45.1	<0.01
SP-A (<43.8/≥43.8 ng/ml)	6/23	7/60	0.15
SP-D (ng/ml)	308 ± 204	258 ± 154	0.40
SP-D (<110/≥110 ng/ml)	4/25	9/58	0.60
KL-6 (U/ml)	894 ± 565	1225 ± 701	<0.01
KL-6 (<500/≥500 U/ml)	8/21	5/62	0.01
GAP index	3.9 ± 1.8	3.6 ± 1.4	0.43

Data presented as the mean ± SD or numbers. FVC = forced vital capacity; FEV1 = forced expiratory volume in 1 s; TLC = total lung capacity; RV = residual volume; DLco = diffusing capacity of the lung for carbon monoxide; SpO_2_ = arterial oxygen saturation measured by pulse oximetry; A-aDO_2_ = alveolar-arterial oxygen difference; ANA = antinuclear antibody; KL-6 = Krebs von den Lungen-6; SP = surfactant protein; GAP = (gender (G), age (A), and two lung physiology variables (P) (FVC and DLco)).

**Table 3:** Radiological findings in IPPFE patients.

HRCT findings	Number
Upper lobe involvement (limited to the upper lobe)	29 (2)
Upper lobe findings	
Subpleural consolidation	29
Honeycombing	9
Traction bronchiectasis	27
Middle or lower lobe involvement	27
Definite UIP pattern	10
Possible UIP pattern	7
NSIP pattern	10

HRCT = high-resolution computed tomography; UIP = usual interstitial pneumonia; NSIP = nonspecific interstitial pneumonia.

**Table 4:** Outcomes and events during the follow-up period.

	IPPFE	IPF	*p* value
	(*n*=29)	(*n*=67)	
Outcome			
Alive	18	38	
Dead	11	29	
Cause			NS
Chronic respiratory failure	4	11	
Acute exacerbation	4	12	
Lung cancer	0	1	
Others	3	5	

Events			
Pneumothorax/ pneumomediastinum	19	4	<0.01
Recurrent infection	2	3	NS

Treatment			NS
Oral corticosteroids	9	17	
Immunosuppressant drugs	2	13	
Pirfenidone	10	31	
Home oxygen therapy	6	26	

**Table 5:** Annual changes in the pulmonary function parameters.

	IPPFE (*n*=17)	IPF (*n*=57)	*p* value
Observation period (years)	1.01 ± 0.19	1.03 ± 0.24	0.59
ΔFVC (L)	–0.28 ± 0.27	–0.11 ± 0.26	0.02
ΔTLC (L)	–0.32 ± 0.26	–0.06 ± 0.39	0.01
ΔRV (L)	–0.04 ± 0.18	–0.07 ± 0.56	0.83
ΔDLco (ml/min/mmHg)	–0.70 ± 1.95	–0.45 ± 1.60	0.87

Data presented as the mean ± SD. Changes in the pulmonary function parameters as assessed at the 1-year follow-up appointment. FVC = forced vital capacity; TLC = total lung capacity; RV = residual volume; DLco = diffusing capacity of the lung for carbon monoxide.

**Table 6:** Prognostic factors for the overall survival of IPPFE patients during the follow-up period.

Parameter	HR (95% CI)	*p* value
*Univariate Cox proportional hazards model*		
Age	0.967 (0.887–1.053)	0.436
Sex (F/M)	4.866 (1.422–16.650)	0.012
Clubbed finger (*p*/*n*)	4.032 (1.098–14.801)	0.036
Fine crackles (*p*/*n*)	1.609 (0.338–7.658)	0.550
APDT/TDT (%)	0.983 (0.892–1.084)	0.737
BMI	0.990 (0.813–1.206)	0.921
Smoking history (*y*/*n*)	3.014 (0.876–10.374)	0.080
Pack-years	1.024 (1.004–1.045)	0.020
History of pneumothorax	7.829 (1.256–48.45)	0.027
GAP index	1.675 (1.153–2.435)	0.007
GAP stages (II + III/I)	10.841 (2.258–52.048)	0.003
SpO_2_ (%)	1.141 (0.708–1.838)	0.588
FVC %pred (%)	0.946 (0.909–0.984)	0.006
FEV_1_/FVC (%)	0.998 (0.954–1.043)	0.912
TLC %pred (%)	0.883 (0.824–0.953)	0.001
RV %pred (%)	0.992 (0.961–1.025)	0.642
RV/TLC %pred (%)	1.001 (0.984–1.018)	0.903
DLco %pred (%)	0.866 (0.799–0.939)	<0.001
DLco/VA %pred (%)	0.895 (0.831–0.964)	0.003
PaCO_2_ (Torr)	1.086 (0.909–1.298)	0.361
A-aDO_2_ (Torr)	1.090 (1.018–1.167)	0.013
ANA (40≥/40<)	1.144 (0.334–3.923)	0.831
SP-A (ng/ml)	1.006 (0.983–1.031)	0.604
SP-D (ng/ml)	1.001 (0.998–1.004)	0.601
KL-6 (U/ml)	1.001 (1.000–1.002)	0.027
ΔFVC (L)	0.016 (0.001–0.433)	0.014
ΔTLC (L)	0.001 (0.000–0.113)	0.005
ΔRV (L)	0.018 (0.000–1.811)	0.088
ΔRV/TLC (%)	1.018 (0.838–1.236)	0.857
ΔDLco (ml/min/mmHg)	0.021 (0.000–50180)	0.607
Lower lobe involvement (definite UIP and possible UIP/NSIP)	2.199 (0.626–7.730)	0.219

*Multivariate Cox proportional hazards model*		
GAP index	2.510 (1.245–5.059)	0.010
FVC %pred (%)	0.903 (0.830–0.982)	0.017

APDT = anteroposterior diameter of the thorax; TDT = transverse diameter of the thorax; BMI = body mass index; GAP = (gender (G), age (A), and two lung physiology variables (P) (FVC and DLco)); SpO_2_ = arterial oxygen saturation measured by pulse oximetry; FVC = forced vital capacity; FEV1 = forced expiratory volume in 1 s; TLC = total lung capacity; RV = residual volume; DLco = diffusing capacity of the lung for carbon monoxide; A-aDO_2_ = alveolar-arterial oxygen difference; ANA = antinuclear antibody; SP = surfactant protein; KL-6 = Krebs von den Lungen-6.
